# Immunoinformatics based design of a multiepitope vaccine targeting *Streptobacillus moniliformis* for the prevention of rat-bite fever

**DOI:** 10.1515/med-2026-1431

**Published:** 2026-06-05

**Authors:** Muhammad Naveed, Muhammad Toheed, Tariq Aziz, Muhammad Asim, Hafiz Muzzammel Rehman, Nausheen Nazir, Manal F. Elkhadragy, Rania Ali El Hadi Mohamed, Ashwag Shami, Maher S. Alwethaynani, Deema Fallatah

**Affiliations:** Department of Biotechnology, Faculty of Science and Technology, University of Central Punjab, Lahore, Pakistan; School of Biomedical Engineering, Shenzhen University, Shenzhen, Guangdong, China; School of Biochemistry & Biotechnology, University of the Punjab, Lahore, Pakistan; Department of Biochemistry, University of Malakand, Chakdara, Pakistan; Department of Biology, College of Science, Princess Nourah bint Abdulrahman University, Riyadh, Saudi Arabia; Department of Clinical Laboratory Sciences, College of Applied Medical Sciences, Shaqra University, Alquwayiyah, Riyadh, Saudi Arabia; Department of Medical Laboratory Sciences, College of Applied Medical Sciences, Prince Sattam Bin Abdulaziz University, Al-Kharj, Saudi Arabia

**Keywords:** *Streptobacillus moniliformis*, rat-bite fever, multiepitope vaccine, immunoinformatics, molecular docking, molecular dynamics simulation

## Abstract

**Objectives:**

To design and computationally evaluate a multiepitope-based vaccine candidate targeting antigenic proteins of *Streptobacillus moniliformis* using immunoinformatics approaches.

**Methods:**

Three antigenic proteins, ATP-dependent zinc metalloprotease FtsH, signal peptidase I, and protein translocase subunit SecY, were selected based on antigenicity, non-allergenicity, and non-toxicity. B-cell and T-cell epitopes were predicted and screened for immunogenic potential and global population coverage. A multiepitope vaccine construct containing six MHC class I and six MHC class II epitopes was assembled using appropriate linkers and a beta-defensin-3 adjuvant. Structural modeling, molecular docking with Toll-like receptor 4 (TLR4), and molecular dynamics simulations were performed to assess structural behavior and receptor interactions. Codon optimization and *in silico* cloning were conducted to evaluate expression feasibility. Immune simulations were performed to estimate potential immune responses.

**Results:**

The final vaccine construct showed antigenicity with a VaxiJen score of 0.5666 and structural stability with an instability index of 32.50. Population coverage analysis indicated 93.20 % global coverage. Molecular docking and molecular dynamics simulations indicated stable interactions with TLR4. Codon optimization suggested improved translational compatibility in *Escherichia coli*. Immune simulation indicated increased levels of IgG and IgM antibodies and cytokines including IFN gamma and IL-2.

**Conclusions:**

The computational analysis suggests that the designed multiepitope construct has characteristics associated with immunogenic potential and structural stability. Further experimental validation is required to confirm its biological efficacy.

## Introduction

Rat-bite fever (RBF) is a zoonotic disease caused by *Streptobacillus moniliformis*, a Gram-negative, filamentous bacterium that forms part of the normal flora of the respiratory tract and oral cavity of rodents [1]. The infection is primarily transmitted to humans through bites or scratches from infected rodents, including rats, mice, and gerbils, or through contact with contaminated food, water, or surfaces [2]. Following an incubation period of 3–10 days, infected individuals typically develop symptoms such as fever, chills, headache, myalgia, and polyarthritis, often accompanied by a characteristic maculopapular or petechial rash [3]. In severe cases, RBF may result in life-threatening complications, including endocarditis, meningitis, pneumonia, and septic arthritis, particularly in immunocompromised individuals or those with delayed diagnosis and treatment [4]. The nonspecific nature of the early clinical manifestations may complicate timely diagnosis and disease management [5].

Rat-bite fever is a globally distributed zoonotic disease; however, its true prevalence is likely underestimated due to underreporting, misdiagnosis, and limited awareness among healthcare providers [6]. High rodent densities and close human–rodent contact in urban and informal settlements with poor sanitation may increase the public health risk of rat-bite fever transmission [7]. Certain occupational groups, including laboratory personnel, pet store workers, veterinarians, and individuals residing in rodent-infested environments, are at increased risk of infection due to greater exposure to rodents or contaminated materials [8]. Children and pet owners who handle rodents are also vulnerable, as transmission may occur through bites, scratches, or direct contact with infected animals, even in the absence of visible injuries [9]. Despite its widespread distribution, RBF is often overlooked in differential diagnoses, resulting in delayed treatment and a higher risk of serious complications [10].

Currently, the primary treatment for rat-bite fever caused by *S. moniliformis* relies on antibiotic therapy, with penicillin considered the drug of choice due to its high efficacy against the pathogen [11]. In patients with penicillin allergy, alternative agents such as doxycycline, streptomycin, or erythromycin may be administered [12]. Appropriate treatment and control measures are important for reducing the burden and complications associated with rat-bite fever [13]. However, diagnostic challenges and limitations in the identification and management of Streptobacillus infections may complicate effective treatment and disease control [14]. These limitations underscore the need for preventive strategies that reduce dependence on antimicrobial therapy.

*Streptobacillus moniliformis* possesses multiple virulence factors that facilitate infection and immune evasion. The bacterium adheres to host tissues through specific adhesion proteins that promote colonization and invasion [15]. Infection with *S. moniliformis* is associated with inflammatory responses that may contribute to tissue damage and systemic manifestations such as fever and arthralgia [16]. In addition, biofilm formation can enhance microbial persistence by protecting bacteria from host immune responses and environmental stressors [17]. Collectively, these pathogenic mechanisms contribute to severe systemic complications, particularly in immunocompromised individuals or in cases of delayed diagnosis [18].

In light of the absence of an approved vaccine and the growing concern of antimicrobial resistance, there is a pressing need to explore preventive strategies against *S. moniliformis*. The objective of this study was to design and evaluate a multiepitope-based *in silico* vaccine candidate using immunoinformatics approaches. Highly immunogenic epitopes derived from key virulence-associated proteins were identified and selected based on antigenicity, non-allergenicity, and their potential to induce both humoral and cellular immune responses. We hypothesized that a rationally constructed multiepitope vaccine could demonstrate structural stability, effective immune receptor interaction, and favorable predicted immunogenicity. Accordingly, the research question addressed was whether an immunoinformaticsguided vaccine design could generate a stable and immunogenic candidate against *S. moniliformis*. This computational strategy provides a foundation for future experimental validation and vaccine development.

## Materials and methods

### Protein selection

The protein sequences of *S. moniliformis* were retrieved from the UniProt database (https://www.uniprot.org/) in FASTA format. Subcellular localization and the presence of transmembrane helices were predicted using TMHMM version 2.0 (https://services.healthtech.dtu.dk/services/TMHMM-2.0/) to identify membrane-associated proteins [19]. The selected proteins were further evaluated for antigenicity using VaxiJen version 2.0 (https://www.ddg-pharmfac.net/vaxijen/VaxiJen/VaxiJen.html), applying the default threshold for bacterial models [20]. Allergenicity was assessed using AllerTOP version 2.1 (https://www.ddg-pharmfac.net/allertop_test/) to ensure the selection of non-allergenic candidates [21].

### T-cell epitope prediction

MHC class I epitopes were predicted using the ANN 4.0 algorithm available on the IEDB server (http://tools.iedb.org/mhci/). Epitope selection was based on binding affinity, with IC50 values below 100 nM considered indicative of strong binding to MHC class I molecules. MHC class II epitopes were predicted using the NN-align method implemented in the IEDB server (http://tools.iedb.org/mhcii/), which integrates position-specific scoring matrices with machine learning approaches. A selection threshold of IC50 values lower than 100 nM was applied for MHC class II binding predictions [22], 23].

### Population coverage analysis

To estimate the potential global applicability of the predicted T-cell epitopes, population coverage analysis was performed using the IEDB Population Coverage tool (http://tools.iedb.org/population/) [24]. The identified epitopes along with their associated HLA alleles were submitted to the platform to calculate the proportion of individuals in various geographic regions and ethnic groups who may respond to the selected epitopes.

### Epitope evaluation

All predicted epitopes were further screened to ensure their suitability for vaccine development. Antigenicity was assessed using the VaxiJen version 2.0 server. Allergenicity prediction was performed with AllerTOP version 2.1 to exclude potentially allergenic peptides. Toxicity analysis of the selected epitopes was conducted using the ToxinPred server (https://crdd.osdd.net/oscadd/toxipred/) [25]. Only epitopes meeting the criteria of being antigenic, non-allergenic, and non-toxic were retained for subsequent vaccine construction.

### Vaccine construct design

The multiepitope vaccine construct was designed by sequentially linking the selected epitopes using appropriate peptide linkers. MHC class I epitopes were connected using AAY linkers to facilitate proteasomal processing and enhance cytotoxic T-lymphocyte responses. MHC class II epitopes were joined with SSL linkers to support proper structural conformation and improve helper T-cell activation. To enhance immunogenicity, beta-defensin-3 was incorporated at the N-terminal region of the construct and linked to the first MHC class I epitope through an AAY linker. Beta-defensin-3 was selected as an adjuvant due to its well-documented immunomodulatory properties, including activation of antigen-presenting cells, enhancement of dendritic cell maturation, and promotion of T-cell–mediated immune responses. Moreover, beta-defensin-3 has been reported to stimulate innate immune pathways and improve antigen presentation, thereby increasing the overall immunogenic potential of multiepitope vaccine constructs. A 6× histidine tag was appended to the C-terminal end to facilitate purification and detection of the expressed protein. The final vaccine construct was subsequently evaluated for antigenicity and allergenicity to confirm its suitability for further analysis [26].

### Physicochemical properties analysis

The physicochemical properties of the final multiepitope vaccine construct were analyzed using the ExPASy ProtParam tool (https://web.expasy.org/protparam/) [27], 28]. This server calculates key parameters based on the provided amino acid sequence. The evaluated properties included molecular weight, theoretical isoelectric point, instability index, aliphatic index, grand average of hydropathicity (GRAVY), and predicted half-life. Additionally, the solubility of the designed vaccine construct upon overexpression was predicted using SoluProt v1.0 (https://loschmidt.chemi.muni.cz/soluprot/) to assess its potential for soluble expression in *Escherichia coli* [29].

### Secondary structure prediction

The PSIPRED (https://bioinf.cs.ucl.ac.uk/psipred/) and SOPMA (https://npsa.lyon.inserm.fr/cgi-bin/npsa_automat.pl?page=/NPSA/npsa_sopma.html) servers were used for predicting the secondary structure of the vaccine construct. The amino acid sequence of the construct was submitted to both platforms to estimate the proportion of α-helices, β-strands, and random coils. PSIPRED employs a neural network–based approach for structure prediction, whereas SOPMA utilizes alignment-based algorithms to determine secondary structural elements [30].

### Tertiary structure prediction

The three-dimensional structure of the designed vaccine construct was predicted using AlphaFold3 (https://alphafoldserver.com/), a deep learning–based algorithm developed for protein structure modeling [31]. The predicted model was subsequently evaluated for structural quality and reliability using the PROCHECK and ERRAT validation tools available through the SAVES server (https://saves.mbi.ucla.edu/) [32], 33]. PROCHECK was employed to generate Ramachandran plots to assess the stereochemical quality of the model by analyzing backbone dihedral angles. ERRAT was used to evaluate the overall structural integrity by examining non-bonded atomic interactions and identifying potentially misfolded regions within the predicted structure.

### Discontinuous B-cell epitope prediction

Discontinuous B-cell epitopes were predicted using the Ellipro tool accessible on the IEDB server (http://tools.iedb.org/ellipro/). The vaccine construct PDB file was uploaded and default settings of a minimum score of 0.5 and a maximum distance of six Å were utilized. ElliPro determines conformational epitopes by calculating residual protrusion index, that measures how much a residue sticks out from the surface of a molecular structure. The algorithm generated scores and residue positions for each predicted epitope [34].

### Molecular docking and interaction analysis

Molecular docking between the vaccine construct and the selected immune receptor was performed using the ClusPro 2.0 protein docking server (https://cluspro.bu.edu/login.php) [35]. Toll-like receptor 4 (TLR4) was selected for docking analysis due to its pivotal role in recognizing Gram-negative bacterial components, particularly lipopolysaccharides (LPS), which are characteristic components of Gram-negative bacteria [36]. Activation of TLR4 triggers MyD88-dependent and TRIF-dependent signaling pathways, leading to NF-κB activation and production of pro-inflammatory cytokines [37]. Additionally, the incorporation of beta-defensin-3 as an adjuvant in the vaccine construct further justifies the selection of TLR4, as beta-defensin-3 has been reported to enhance immune responses via TLR4-mediated pathways [38]. Therefore, TLR4 was considered the most a biologically relevant innate immune receptor for evaluating vaccine–receptor interactions.

The three-dimensional structures of both the receptor and the vaccine construct were prepared in PDB format and submitted to the ClusPro server. The docking algorithm generated multiple conformational poses, which were ranked according to their predicted energy scores. Interaction analysis of the selected vaccine–TLR4 complex was conducted using the PDBsum server (https://www.ebi.ac.uk/thornton-srv/databases/pdbsum/Generate.html) [39]. This analysis identified hydrogen bonds, hydrophobic contacts, and other non-covalent interactions between the vaccine construct and the receptor. Two-dimensional interaction diagrams were generated to illustrate the key amino acid residues involved in binding and to assess the stability of the predicted complex.

### Molecular dynamics simulations

Molecular dynamics simulations were conducted using the Desmond module developed by D. E. Shaw Research to examine the structural behavior of the vaccine–TLR4 complex [40]. The protein structure was prepared using the Protein Preparation Wizard, where bond orders were assigned, hydrogen atoms were added, and appropriate disulfide bonds were defined to ensure structural consistency. The system was solvated in an orthorhombic box with a 10 Å buffer distance using the simple point charge water model [41]. To mimic physiological conditions, sodium chloride was added at a concentration of 0.15 M, and counterions were introduced to neutralize the system. Energy minimization was performed prior to simulation under periodic boundary conditions. Production simulations were carried out for 100 ns using the OPLS4 force field at a temperature of 300 K and a pressure of 1 atm. Electrostatic interactions were treated using the particle mesh Ewald (PME) method with a short-range cutoff distance of 9.0 Å. Temperature and pressure were regulated using the Nosé–Hoover thermostat and the Martyna–Tuckerman–Klein barostat, respectively [42]. System stability and conformational behavior were assessed by calculating root mean square deviation (RMSD) and root mean square fluctuation (RMSF) values. Principal component analysis (PCA) was performed to evaluate dominant collective motions within the complex. Dynamic cross-correlation matrix (DCCM) analysis was conducted to examine correlated atomic movements. In addition, the free energy landscape (FEL) was generated to explore the conformational states sampled during the simulation period [43].

### Immune simulation

Immune response simulation was performed using the C-ImmSim server (https://kraken.iac.rm.cnr.it/C-IMMSIM/index.php), which models antigen-driven adaptive immune responses. The simulation was initialized with a random seed value of 12345, a simulation volume of 10, and a total of 100 time steps. A single vaccine dose was administered at the initial time step without lipopolysaccharide supplementation. The construct was co-administered with the adjuvant at a concentration of 100, and the system included 1,000 antigen units. During the simulation period, the platform modeled key immune processes, including B-cell and T-cell activation and antibody production.

Cytokine responses, including interferon gamma, interleukin 2, interleukin 10, and interleukin 6, were evaluated based on their relative magnitude, temporal patterns, and peak levels in comparison with baseline simulation values generated by the platform. As the C-ImmSim server does not define fixed experimental cut-off thresholds, immune activation was interpreted through comparative trends and dynamic response patterns across the simulation timeline. The simulation outputs were analyzed to estimate the potential immunogenic profile of the vaccine construct and to characterize the predicted involvement of immune cell populations during the simulated response [44].

### Codon optimization and *in silico* cloning

The amino acid sequence of the designed vaccine construct was reverse translated into a nucleotide sequence using the EMBOSS backTranSeq tool (https://www.ebi.ac.uk/jdispatcher/st/emboss_backtranseq) [45]. The resulting nucleotide sequence was subsequently optimized to enhance its expression efficiency in the selected host organism. Codon optimization was performed using the NovoPro codon optimization tool (ExpoOptimizer) (https://www.novoprolabs.com/tools/codon-optimization), which adapts the nucleotide sequence according to the codon usage preferences of the host to improve translational efficiency and protein expression [46]. Following optimization, the adapted gene sequence was inserted into the pET-29a(+) expression vector using SnapGene for *in silico* cloning. The optimized construct was positioned within the vector to ensure correct orientation and reading frame alignment. Promoter regions and restriction sites were verified to confirm compatibility with the pET-29a(+) system and suitability for potential expression in *E. coli* K12.

### Ethical approval

Not applicable.

### Clinical trial number

Not applicable.

## Results

### Retrieval and selection of *S. moniliformis* proteins

Based on antigenicity scores and predicted non-allergenicity, three proteins of *S. moniliformis* were selected for further analysis. The details of the selected proteins, including their UniProt accession numbers, are presented in Table 1.

**Table 1:** List of selected proteins of *Streptobacillus moniliformis*.

No.	Uniprot ID	Protein names	Antigenicity	Allergenicity
1.	D1AXT4	ATP-dependent zinc metalloprotease FtsH	0.5836	Non-allergen
2.	D1AWV0	Signal peptidase I	0.4448	Non-allergen
3.	D1AVF8	Protein translocase subunit SecY	0.4790	Non-allergen

**Figure 1:**
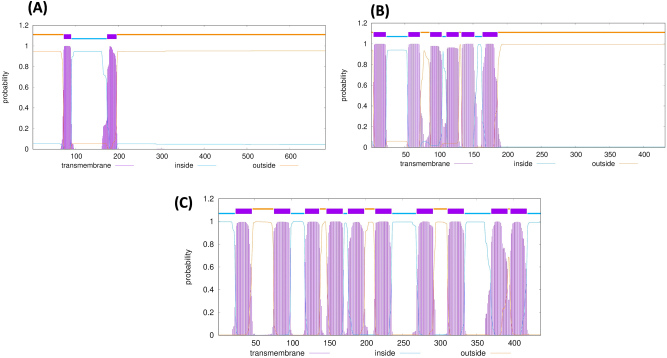
Graphical representation of the predicted transmembrane regions of the selected proteins using the TMHMM server **(A)** ATP-dependent zinc metalloprotease FtsH. **(B)** Signal peptidase I. **(C)** Protein translocase subunit SecY.

Subcellular localization and the presence of transmembrane helices were predicted using the TMHMM server. The predicted membrane topology of each selected protein is illustrated in Figure 1.

### T-cell epitope prediction

MHC class I epitopes were predicted from the three selected proteins based on a binding affinity threshold of IC50 less than 100 nM. Two high-affinity epitopes along with their corresponding HLA alleles were identified for each protein. The selected epitopes were evaluated based on antigenicity, allergenicity, toxicity, HLA-binding affinity, and population coverage analyses. While one selected epitope, EAAILAARK, exhibited comparatively lower predicted antigenicity, it was retained due to its favorable HLA-binding affinity, non-allergenicity, non-toxicity, and contribution to broader population coverage. Moreover, the final multiepitope vaccine construct demonstrated overall antigenic potential together with non-allergenic and non-toxic characteristics. Detailed information regarding the selected MHC class I epitopes is presented in Table 2.

**Table 2:** List of selected MHC class I epitopes of targeted proteins.

Protein name	Epitopes	Antigenicity	Allergenicity	Toxicity	Alleles	IC50 score
ATP-dependent zinc metalloprotease FtsH	ILKVHSRNK	1.1413	Non-allergen	Non-toxic	HLA-A*03:01, HLA-A*30:01	68.51
	EAAILAARK	−0.4903	Non-allergen	Non-toxic	HLA-A*68:01	12.68
Signal peptidase I	FIINIMLSI	1.6529	Non-allergen	Non-toxic	HLA-A*02:03, HLA-A*02:01, HLA-A*02:06 HLA-A*68:02	9.01
	FANNVSYRF	0.8302	Non-allergen	Non-toxic	HLA-B*35:01, HLA-B*53:01, HLA-B*58:01	7.27
Protein translocase subunit SecY	FSFFYTAIVF	0.6385	Non-allergen	Non-toxic	HLA-B*15:01, HLA-A*23:01	13.59
	FTLGCFLVAR	0.9655	Non-allergen	Non-toxic	HLA-A*68:01, HLA-A*33:01, HLA-A*31:01	15.15

MHC class II epitopes were similarly predicted using the predefined selection criteria. For each protein, two epitopes from each protein with IC50 values below 100 nM and antigenicity scores greater than 0.5 were selected along with their associated HLA alleles. All shortlisted epitopes were predicted to be non-allergenic and non-toxic. A comprehensive summary of the selected MHC class II epitopes is provided in Table 3.

**Table 3:** List of selected MHC class II epitopes of targeted proteins.

Protein name	Epitopes	Antigenicity	Allergenicity	Toxicity	Alleles	IC50 score
ATP-dependent zinc metalloprotease FtsH	DVKLEDIAKITPGFV	1.2698	Non-allergen	Non-toxic	HLA-DRB1*13:02	38.1
	EDIAKITPGFVGADL	0.4038	Non-allergen	Non-toxic	HLA-DRB1*01:01, HLA-DRB1*09:01, HLA-DRB1*13:02	55.2
Signal peptidase I	FANNVSYRFKSPQIG	0.7584	Non-allergen	Non-toxic	HLA-DRB1*07:01	56.1
	EPTIKVGDRIFANNV	0.6323	Non-allergen	Non-toxic	HLA-DRB1*03:01	45
Protein translocase subunit SecY	ASIFSLGITPYINAS	0.7797	Non-allergen	Non-toxic	HLA-DRB1*09:01, HLA-DRB1*01:01, HLA-DRB1*04:01, HLA-DRB1*15:01	8.4
	FASMIMAVPSAIIPL	0.5789	Non-allergen	Non-toxic	HLA-DRB1*09:01, HLA-DRB1*13:02, HLA-DRB1*01:01, HLA-DRB1*15:01, HLA-DRB1*07:01, HLA-DRB1*08:02, HLA-DRB1*04:01	6.5

### Population coverage analysis

**Figure 2:**
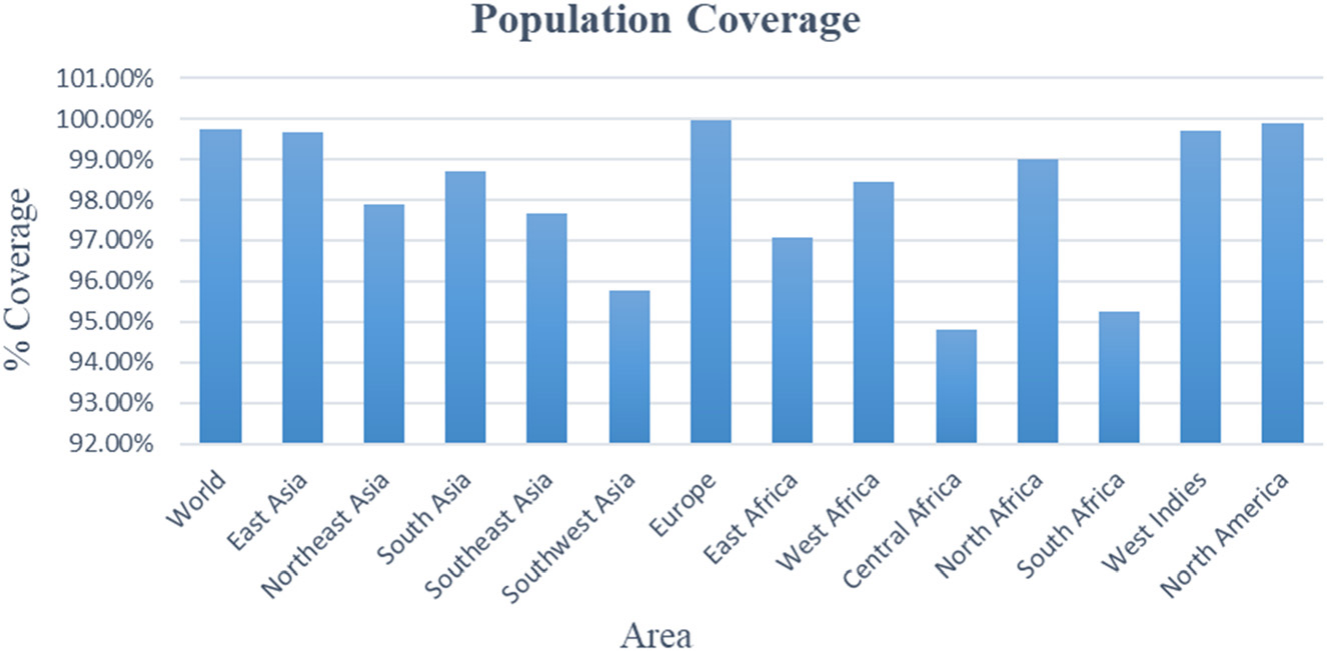
Population coverage analysis of selected T-cell epitopes.

Population coverage analysis of the selected T-cell epitopes demonstrated an estimated global coverage exceeding 99 %. The highest predicted coverage was observed in Europe, East Asia, and North America. Regions such as Central Africa and the West Indies showed slightly lower coverage values of approximately 94 %. Other regions, including South Asia, Southeast Asia, and South Africa, demonstrated coverage values of up to 97 %, as illustrated in Figure 2. These findings reflect the predicted distribution of the selected HLA-binding epitopes across different geographic regions.

### Vaccine construct design

**Figure 3:**
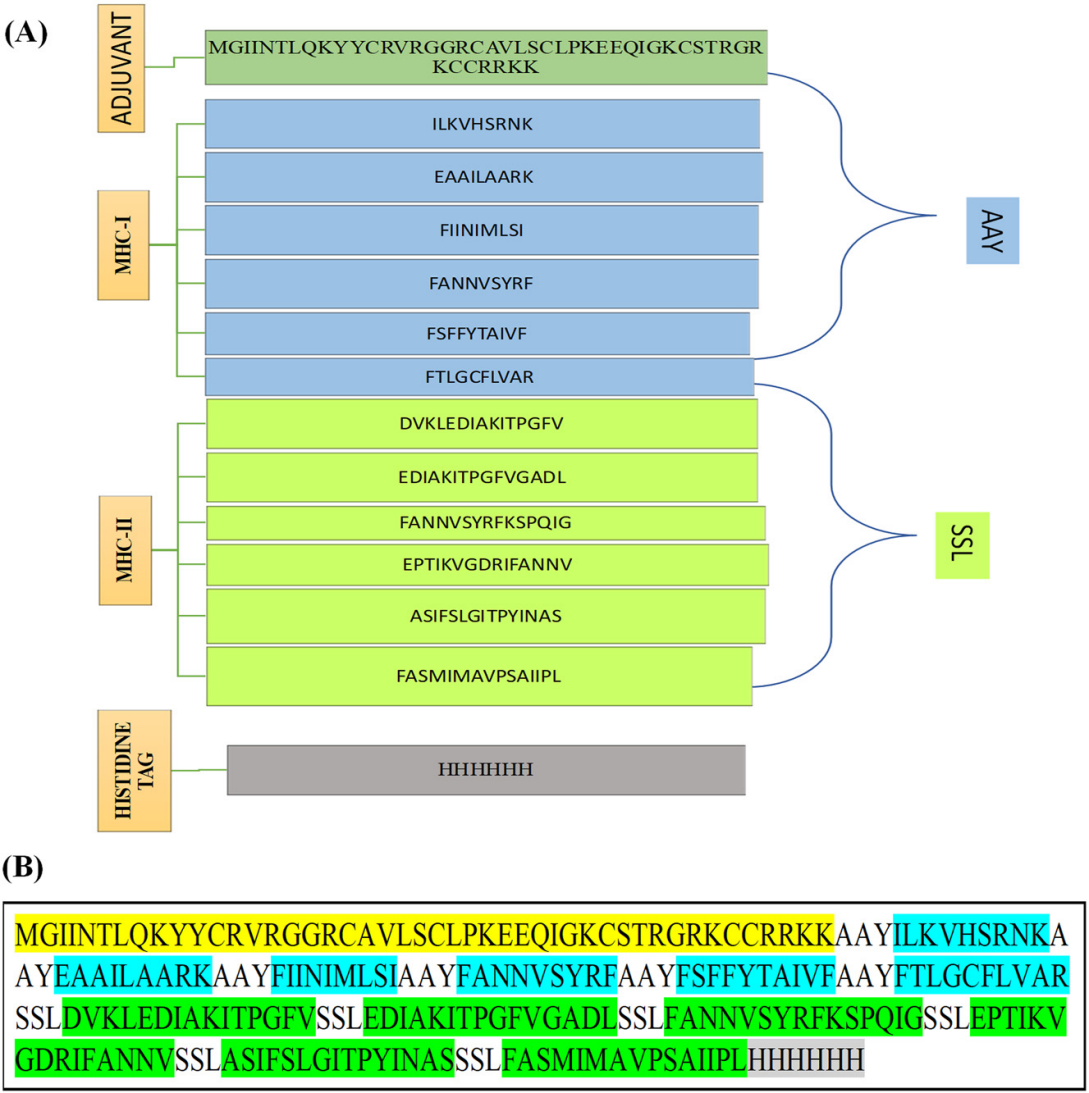
Design of the multiepitope vaccine construct **(A)** graphical representation of the arrangement of vaccine components. **(B)** Complete amino acid sequence of the designed vaccine construct comprising 234 residues. Color coding: beta-defensin-3 adjuvant (yellow), MHC class I epitopes (blue), MHC class II epitopes (green), and linker regions (uncolored).

The final vaccine construct was assembled by linking the selected epitopes using appropriate peptide linkers. Six MHC class I epitopes were joined using AAY linkers, while six MHC class II epitopes were connected using SSL linkers. These linkers were incorporated to facilitate proper structural organization and separation of functional domains within the construct. Beta-defensin-3 was incorporated as an adjuvant at the N-terminal region to enhance the immunogenicity of the vaccine. The schematic organization of the vaccine components is illustrated in Figure 3A, and the complete amino acid sequence of the construct is shown in Figure 3B. Allergenicity analysis using the AllerTOP v2.1 server predicted the construct to be non-allergenic. Antigenicity assessment performed with the VaxiJen v2.0 server yielded a score of 0.5666, exceeding the recommended threshold value of 0.4.

### Physicochemical properties analysis

The physicochemical characteristics of the multiepitope vaccine construct were evaluated based on its amino acid composition. The construct consisted of 234 residues with a calculated molecular weight of 25,746.10 Da and a total of 3,654 atoms. A total of 11 negatively charged residues (Asp + Glu) were present in the sequence. The grand average of hydropathicity value was 0.308, indicating a slightly hydrophobic profile. The instability index suggested that the protein is stable under predicted conditions. The predicted solubility score for expression in *E. coli* was 0.748. A summary of the calculated physicochemical parameters is presented in Table 4.

**Table 4:** Physicochemical properties of the vaccine construct.

Physicochemical properties	
Total number of atoms	3,654
Number of amino acids	234
Molecular weight	25,746.10
Theoretical pI	9.73
Total number of negatively charged residues (Asp + Glu)	11
Total number of positively charged residues (arg + Lys)	27
Estimated half-life	30 h (mammalian reticulocytes, *in vitro*). >20 h (yeast, *in vivo*). >10 h (*Escherichia coli*, *in vivo*)
Instability index	32.50
Grand average of hydropathicity (GRAVY)	0.308
Solubility in *Escherichia coli*	0.748

### Secondary structure prediction

**Figure 4:**
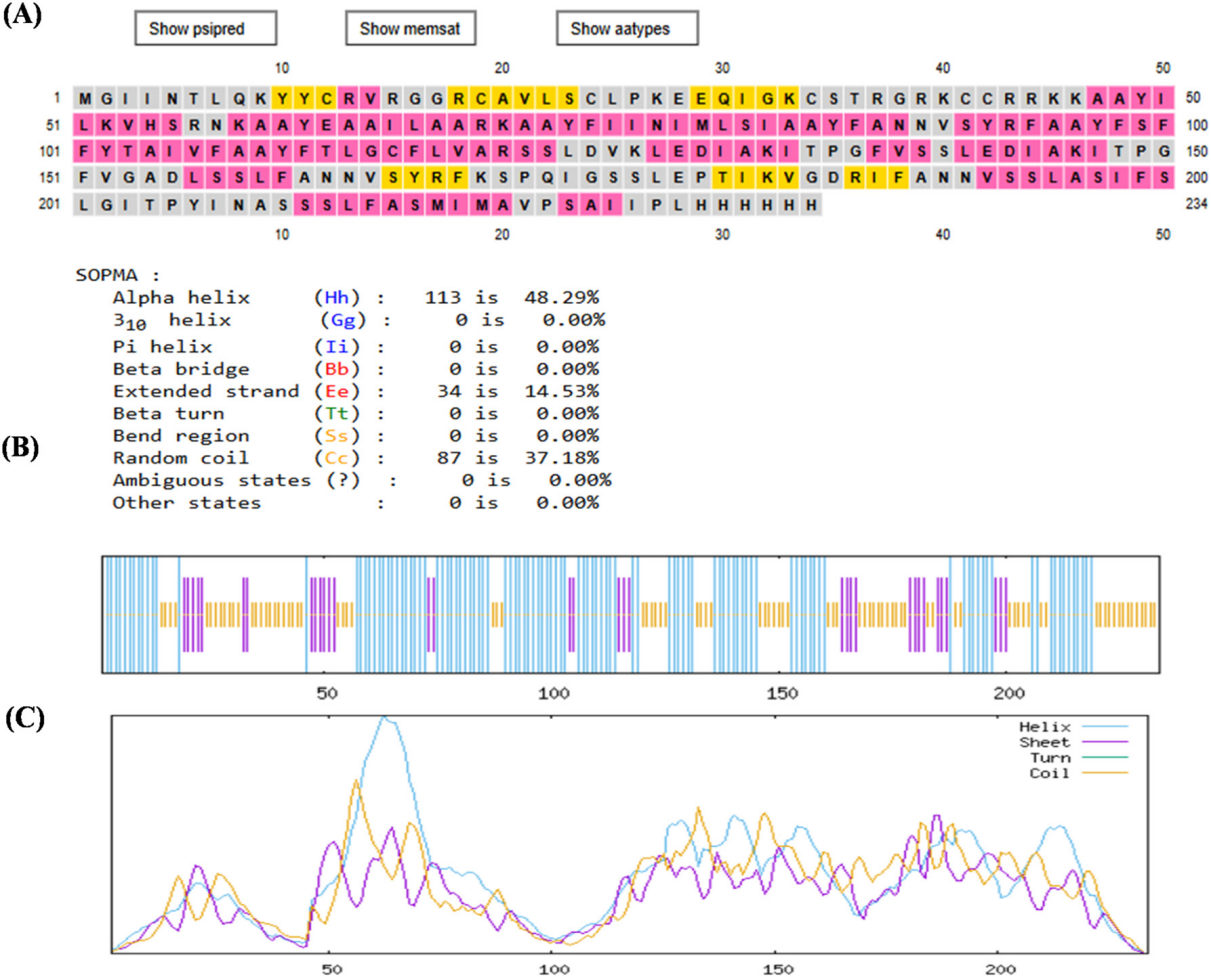
Secondary structure prediction of the vaccine construct. **(A)** PSIPRED prediction showing alpha-helices (pink), beta-strands (yellow), and coils (grey). **(B)** SOPMA representation of the positional distribution and percentage composition of secondary structure elements. **(C)** Graphical distribution of amino acid residues within helix, strand, and coil regions.

The secondary structure of the vaccine construct was predicted using PSIPRED and SOPMA. The analysis indicated that the construct consisted of 48.29 % alpha-helices, 14.53 % beta-strands, and 37.18 % random coils. The PSIPRED prediction is presented in Figure 4A, where alpha-helices are indicated in pink, beta-strands in yellow, and coils in grey. The distribution and positional representation of these secondary structure elements are shown in Figure 4B. Figure 4C illustrates the graphical distribution of amino acid residues within helix, strand, and coil regions of the construct.

### Tertiary structure prediction

**Figure 5:**
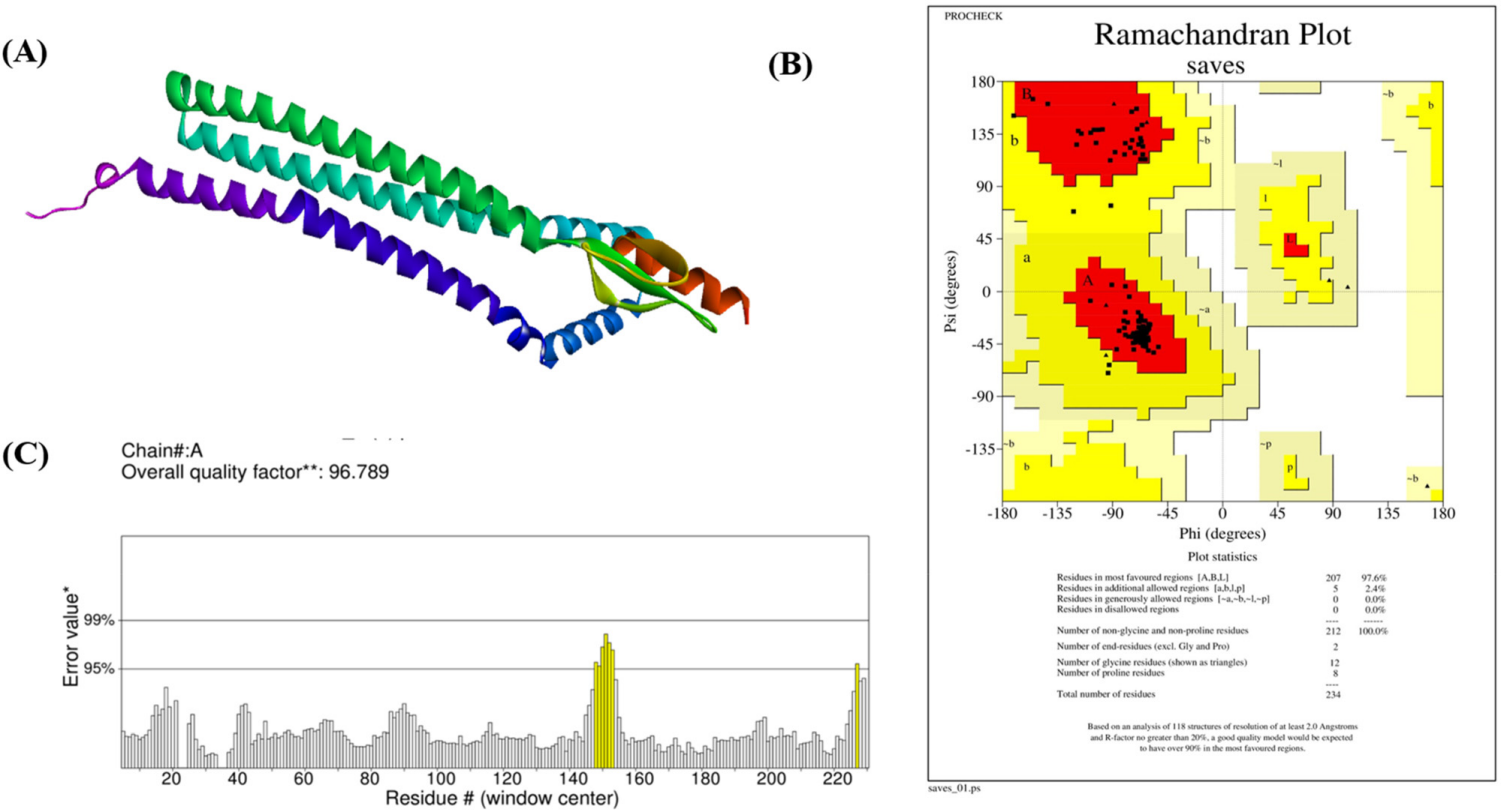
Tertiary structure prediction and validation **(A)** refined three-dimensional structure of the vaccine predicted using GalaxyRefine. **(B)** Ramachandran plot analysis of the refined vaccine structure generated by PROCHECK. **(C)** ERRAT evaluation of the refined vaccine structure.

The vaccine construct’s three-dimensional structure was first predicted using the AlphaFold3 server and further refined using GalaxyRefine, as shown in Figure 5A. Structural validation using the Ramachandran plot showed that 97.6 % of residues were located in the most favored regions and 2.4 % in additionally allowed regions, as presented in Figure 5B. The ERRAT quality assessment yielded an overall quality factor of 96.789, indicating high structural quality. These results demonstrate that the refined vaccine construct exhibits acceptable conformational properties, as illustrated in Figure 5C.

### Discontinuous B-cell epitope prediction

**Figure 6:**
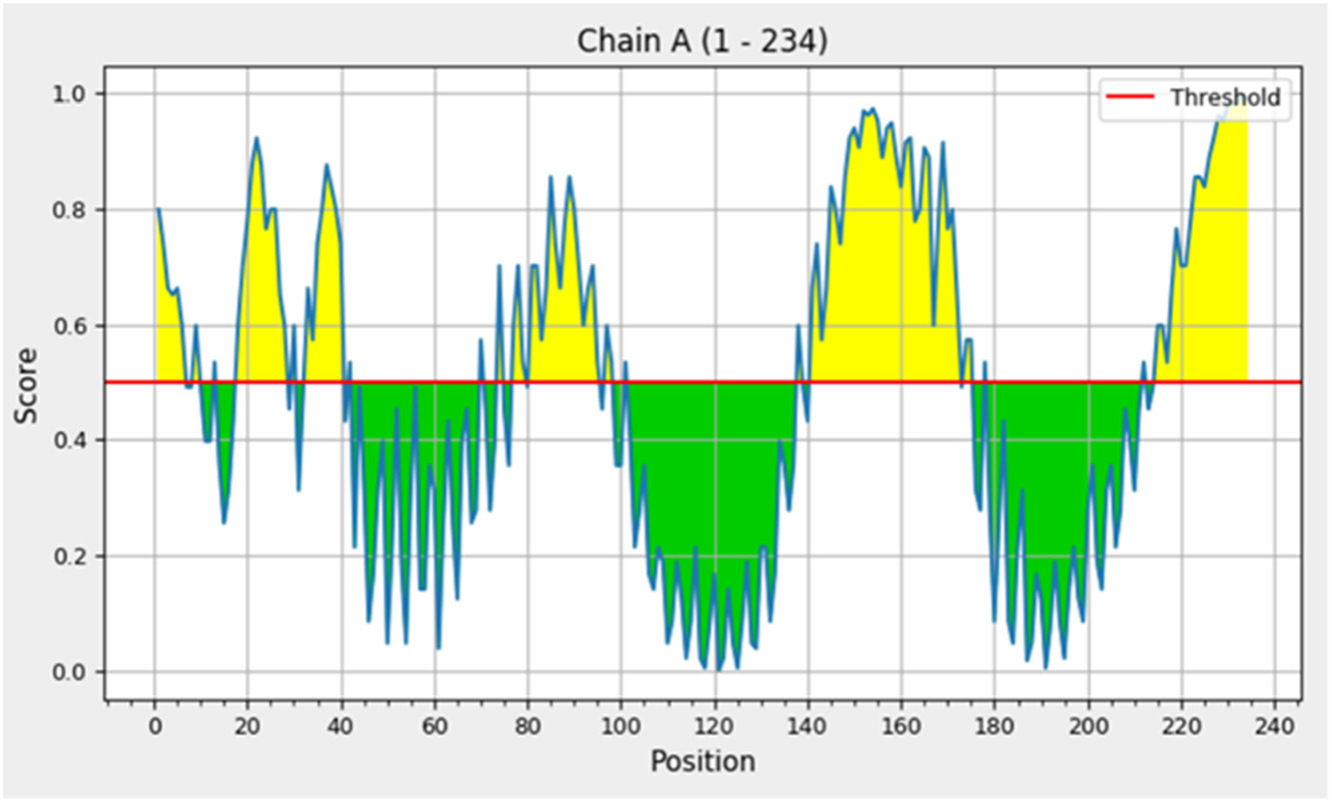
Scores of ElliPro-predicted discontinuous B-cell epitopes of the vaccine construct.

ElliPro identified six discontinuous B-cell epitopes within the vaccine construct, with scores ranging from 0.56 to 0.986, as illustrated in Figure 6. The highest-scoring epitope demonstrated a score of 0.986, indicating strong predicted antigenic potential. The predicted conformational epitopes varied in size and spatial distribution. Epitope 1 comprised 5 residues with a score of 0.986. Epitope 2 included 30 residues with a score of 0.837. Epitope 3 consisted of 3 residues with a score of 0.738. Epitope 4 contained 36 residues with a score of 0.715. Epitope 5 included 29 residues with a score of 0.712, and Epitope 6 comprised 3 residues with a score of 0.56.

**Figure 7:**
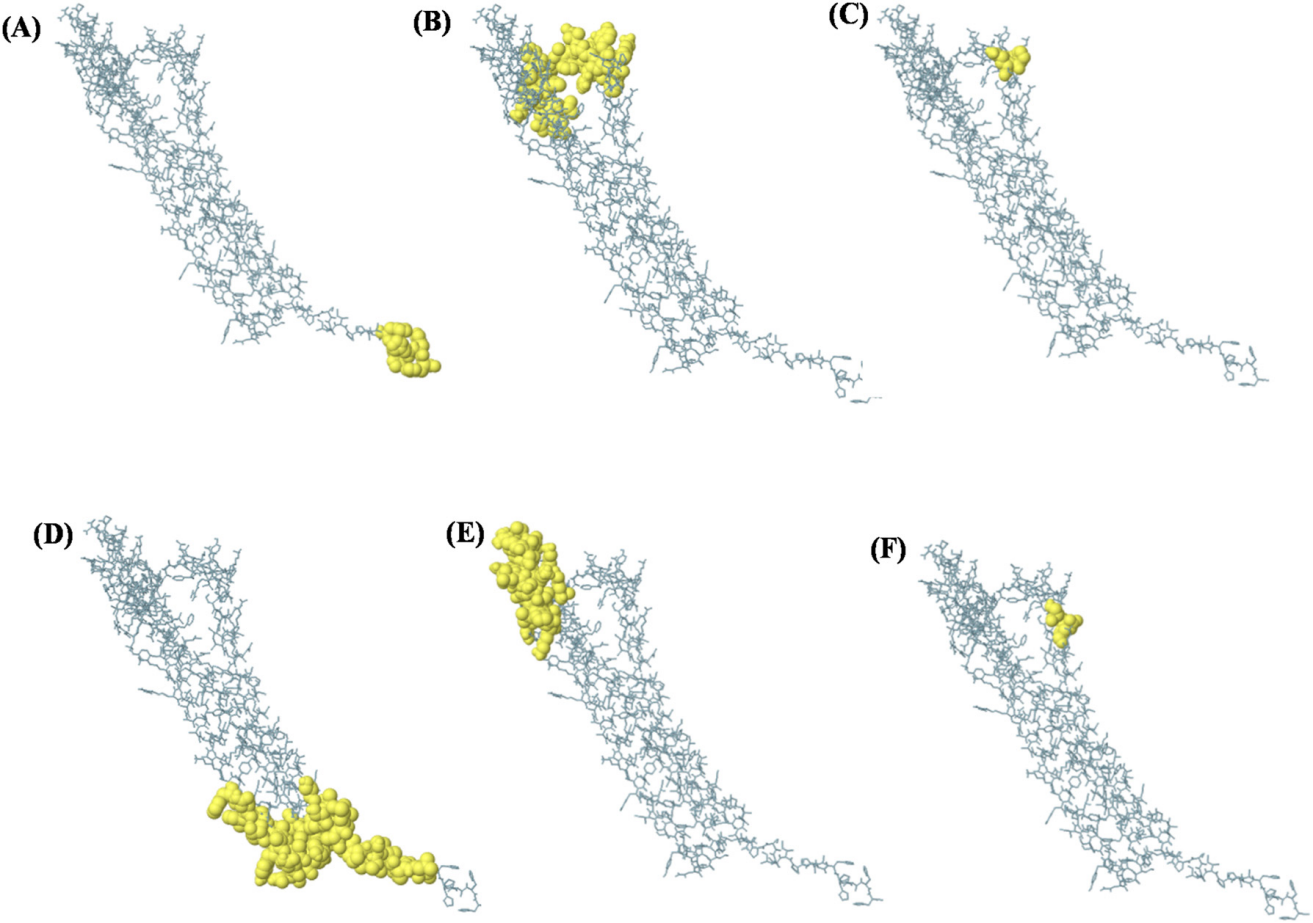
Predicted discontinuous B-cell epitopes (yellow regions) of the designed vaccine construct identified using the ElliPro tool **(A)** Epitope 1 comprising 5 residues with a score of 0.986. **(B)** Epitope 2 comprising 30 residues with a score of 0.837. **(C)** Epitope 3 comprising 3 residues with a score of 0.738. **(D)** Epitope 4 comprising 36 residues with a score of 0.715. **(E)** Epitope 5 comprising 29 residues with a score of 0.712. **(F)** Epitope 6 comprising 3 residues with a score of 0.560.

These epitopes were distributed across different surface-exposed regions of the vaccine construct, including both loop-dominated and structurally ordered regions. The three-dimensional visualization of these predicted epitopes, highlighted in yellow in Figure 7, indicates their accessibility on the protein surface, which is an important characteristic for antibody recognition. Overall, these findings support the presence of multiple predicted conformational B-cell epitopes within the designed vaccine construct, suggesting its potential to induce antibody-mediated immune responses.

### Molecular docking and interactions analysis

**Figure 8:**
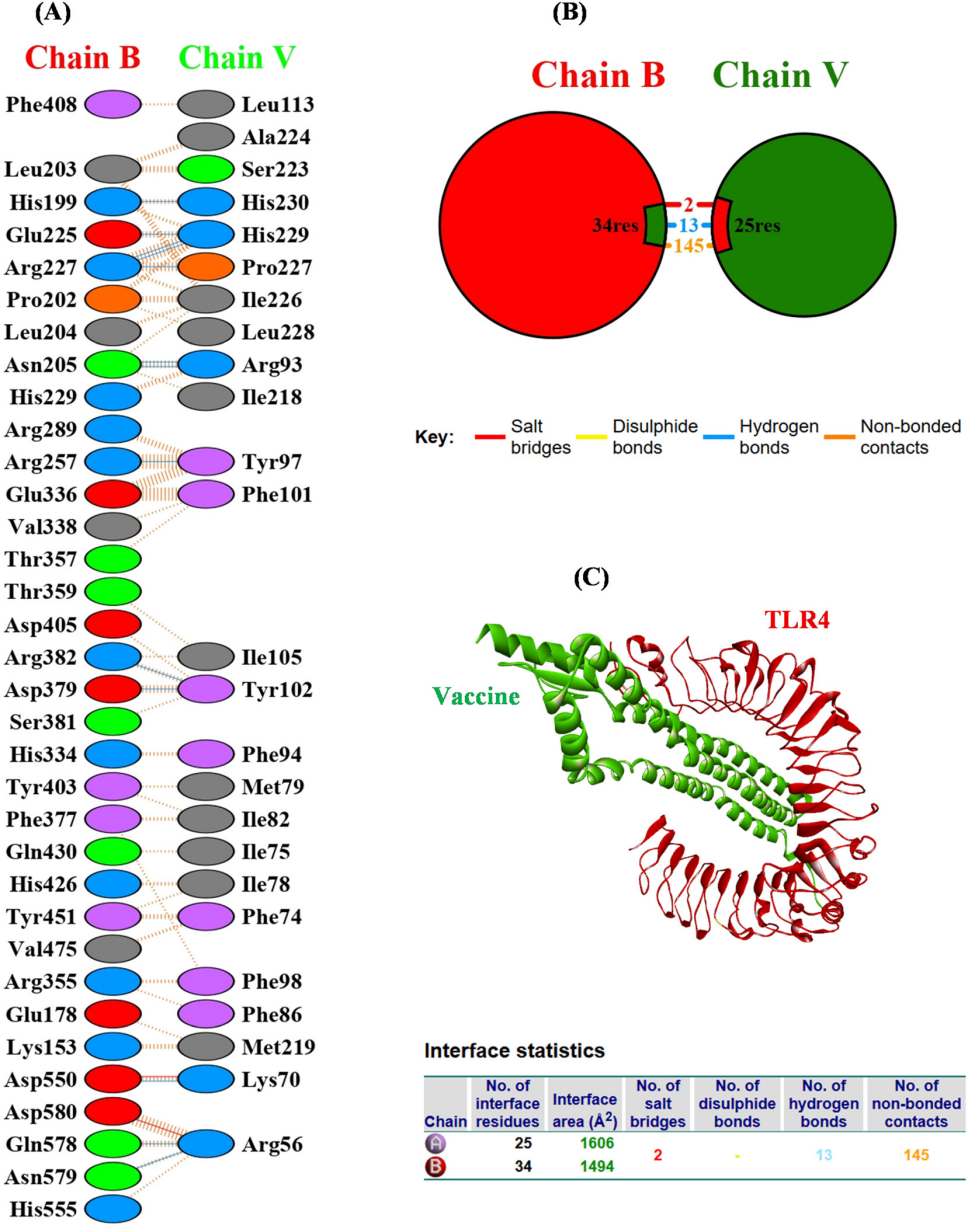
Interaction analysis of the vaccine–TLR4 complex **(A)** residue-level interface interactions showing hydrogen bonds, salt bridges, and non-bonded contacts. **(B)** Interface statistics summarizing interfacial residues and interaction types between TLR4 (Chain B) and the vaccine construct (Chain V). **(C)** Three-dimensional representation of the docked vaccine–TLR4 complex.

The molecular docking analysis of the refined vaccine construct and the TLR4 receptor was performed using the ClusPro server. ClusPro generated 30 different docked conformations, and among these models, the one with the lowest binding energy value of −1312.5 was selected for further evaluation. This model was prioritized based on its energy score and predicted interaction profile, indicating favorable binding between the vaccine construct and TLR4. In the docked complex, Chain V corresponds to the vaccine construct and Chain B corresponds to TLR4. Interaction analysis performed using the PDBsum server revealed multiple stabilizing contacts across the interface. TLR4 contributed 34 interfacial residues, while the vaccine construct contributed 25 residues, corresponding to interface areas of 1,494 Å^2^ and 1,606 Å^2^, respectively, reflecting substantial contact surfaces. The complex exhibited additional stabilization through 2 salt bridges, 13 hydrogen bonds, and 145 non-bonded contacts, including van der Waals contacts, as illustrated in Figure 8.

The hydrogen bond network involved key residue pairs across the interface, including ARG56 with ASN579 and GLN578, LYS70 with ASP550, ARG382 with TYR102, TYR102 with ASP379, ARG257 with TYR97, ARG227 with HIS229 and PRO227, HIS229 with GLU225, ARG93 with ASN205, and HIS230 with HIS199. The hydrogen bond distances ranged from 2.628 Å to 3.182 Å, supporting stable intermolecular interactions. Overall, these interaction features indicate that the designed vaccine construct forms a stable complex with TLR4, an important innate immune receptor. The detailed hydrogen bond interactions predicted by PDBsum are summarized in Table 5.

**Table 5:** Hydrogen bond interactions between the vaccine construct and Chain B of the TLR4 receptor.

Sr. no.	Res. name	Residue no.	Chain		Res. name	Residue name	Chain	Distance (Å)
1	ARG	56	V	–	ASN	579	B	2.870
2	ARG	56	V	–	GLN	578	V	2.936
3	LYS	70	V	–	ASP	550	B	2.628
4	ARG	382	B	–	TYR	102	V	2.661
5	TYR	102	V	–	ASP	379	B	2.832
6	ARG	257	B	–	TYR	97	V	2.754
7	ARG	227	B	–	HIS	229	V	2.973
8	ARG	227	B	–	PRO	227	V	2.765
9	ARG	227	B	–	HIS	229	V	3.182
10	HIS	229	V	–	GLU	225	B	2.712
11	ARG	93	V	–	ASN	205	B	2.681
12	ARG	93	V	–	ASN	205	B	2.629
13	HIS	230	V	–	HIS	199	B	2.907

### Molecular dynamics simulations

**Figure 9:**
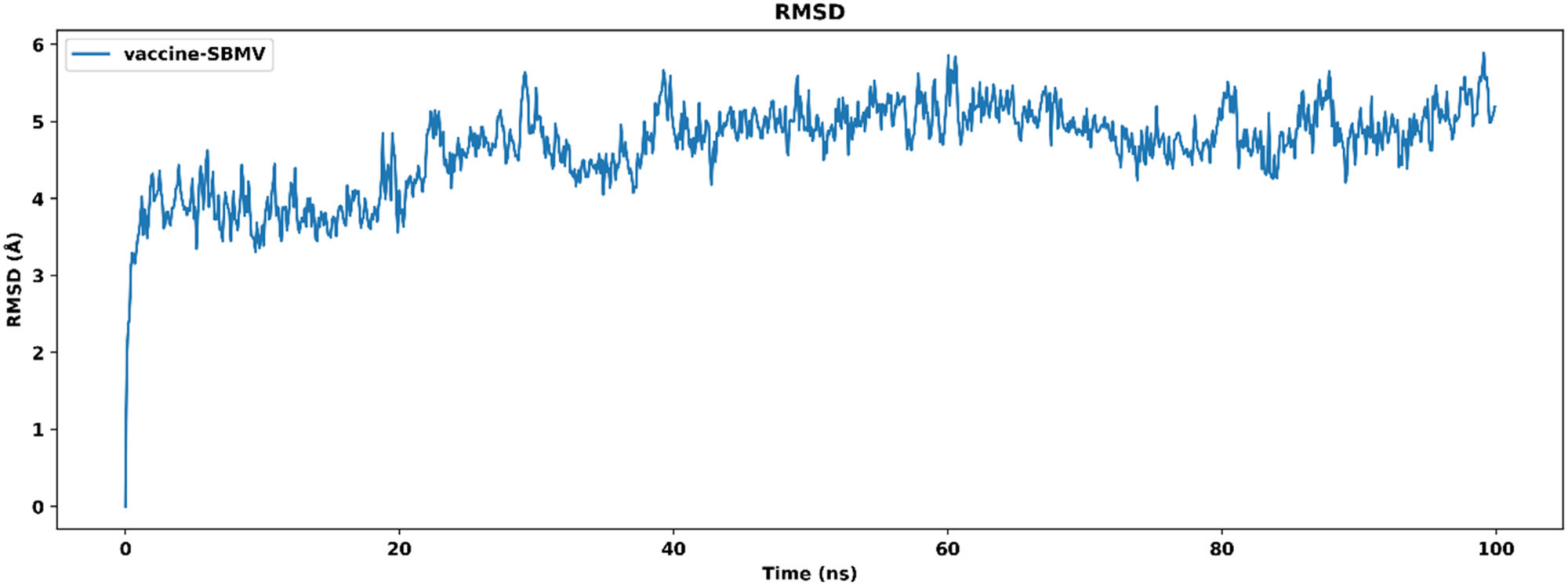
RMSD plot of the vaccine–TLR4 complex over the 100 ns simulation period.

To evaluate the structural behavior of the multiepitope vaccine in complex with TLR4, a 100 ns molecular dynamics simulation was performed. The root mean square deviation of the backbone atoms was monitored throughout the simulation trajectory. The RMSD showed an initial increase during the first 2 ns, rising from 0.0 Å to approximately 3.5 Å, as illustrated in Figure 9. This phase corresponds to early equilibration and conformational adjustment of the system. Following this initial phase, the RMSD gradually increased and began to stabilize around 20 ns, fluctuating within a range of 4.0 Å to 5.5 Å. The average RMSD value between 20 ns and 100 ns was approximately 4.8 Å, reflecting sustained structural stability with moderate flexibility. No major deviations exceeding 6.0 Å were observed during the simulation period, indicating the absence of substantial conformational disruption in the complex.

**Figure 10:**
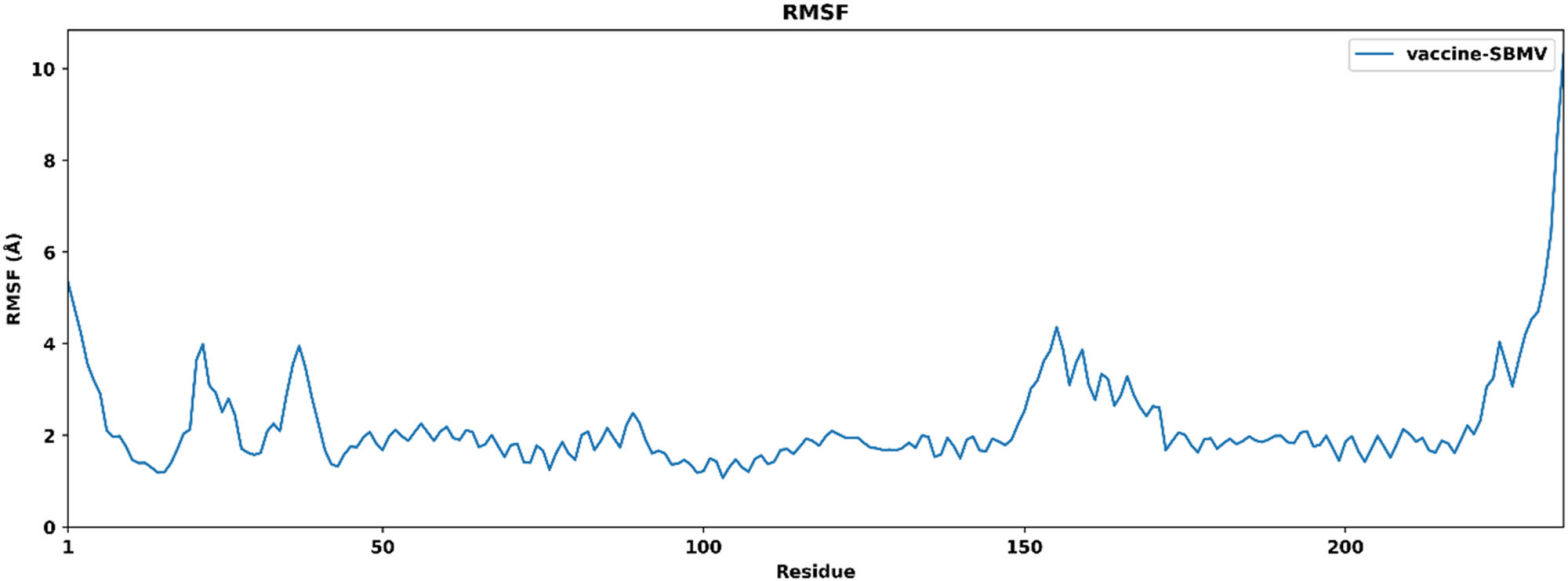
RMSF plot of the vaccine construct showing residue-wise fluctuations during the 100 ns simulation.

RMSF analysis was performed to evaluate the local flexibility of individual residues in the multiepitope vaccine model throughout the 100 ns molecular dynamics simulation. The results showed regions of varying structural mobility, as illustrated in Figure 10. Most residues exhibited relatively low fluctuations, particularly within the region spanning residues 40–180, where RMSF values ranged from 1.0 to 2.5 Å, indicating comparatively stable regions of the structure. These regions correspond to the central portion of the vaccine construct, which includes epitopes and linker segments. In contrast, the N-terminal region (residues 1–20) and the C-terminal region (residues 220–240) displayed higher flexibility, with RMSF values approaching 6.0 Å and exceeding 10.0 Å, respectively. Such elevated fluctuations at terminal regions are commonly observed in protein structures due to reduced structural constraints. Overall, the predominance of low RMSF values across the majority of residues suggests that the vaccine construct maintains structural consistency during the simulation period.

**Figure 11:**
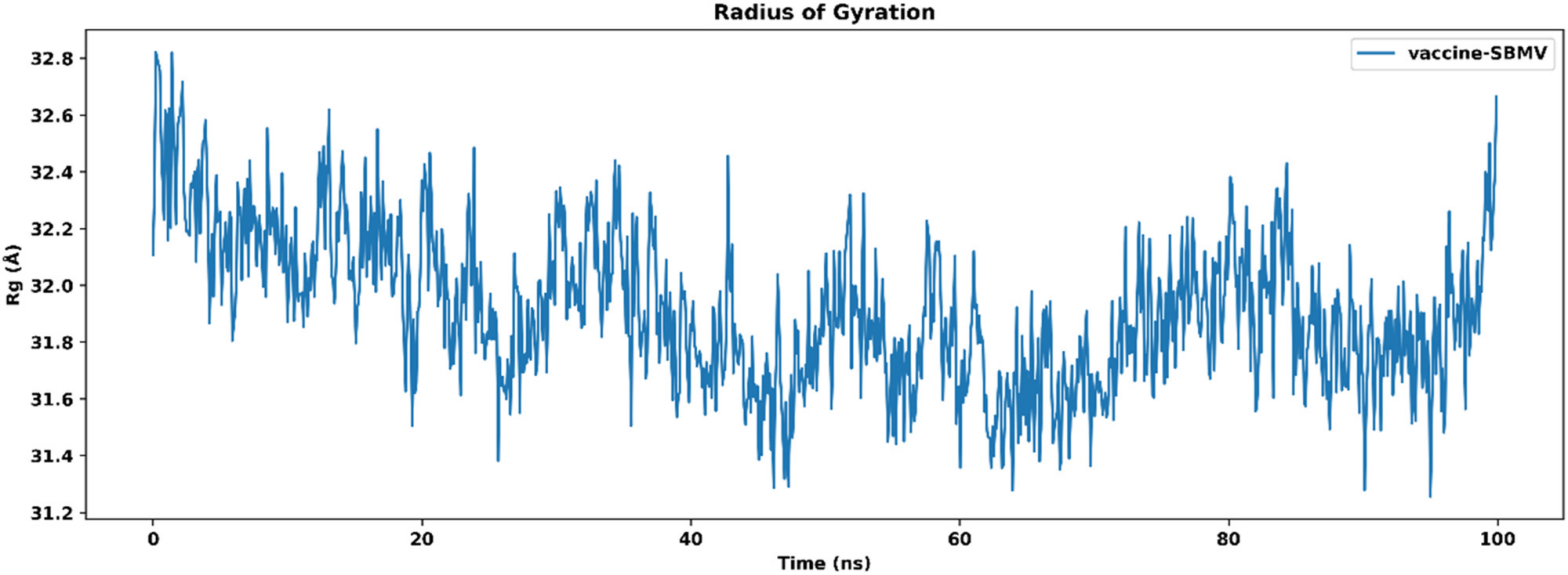
Radius of gyration plot of the vaccine–TLR4 complex during the 100 ns molecular dynamics simulation.

The radius of gyration was evaluated to examine the overall compactness of the vaccine–TLR4 complex during the 100 ns molecular dynamics simulation. The Rg value at the beginning of the simulation was slightly higher at approximately 32.6 Å. Over time, the values gradually decreased and remained relatively stable throughout the simulation period, with minor fluctuations between 31.5 Å and 32.0 Å. The average Rg value was 31.8 Å, as shown in Figure 11. No pronounced peaks or extended deviations were observed in the Rg profile during the simulation, indicating consistent structural behavior of the complex under the simulated conditions. The overall pattern suggests maintenance of structural compactness throughout the trajectory.

**Figure 12:**
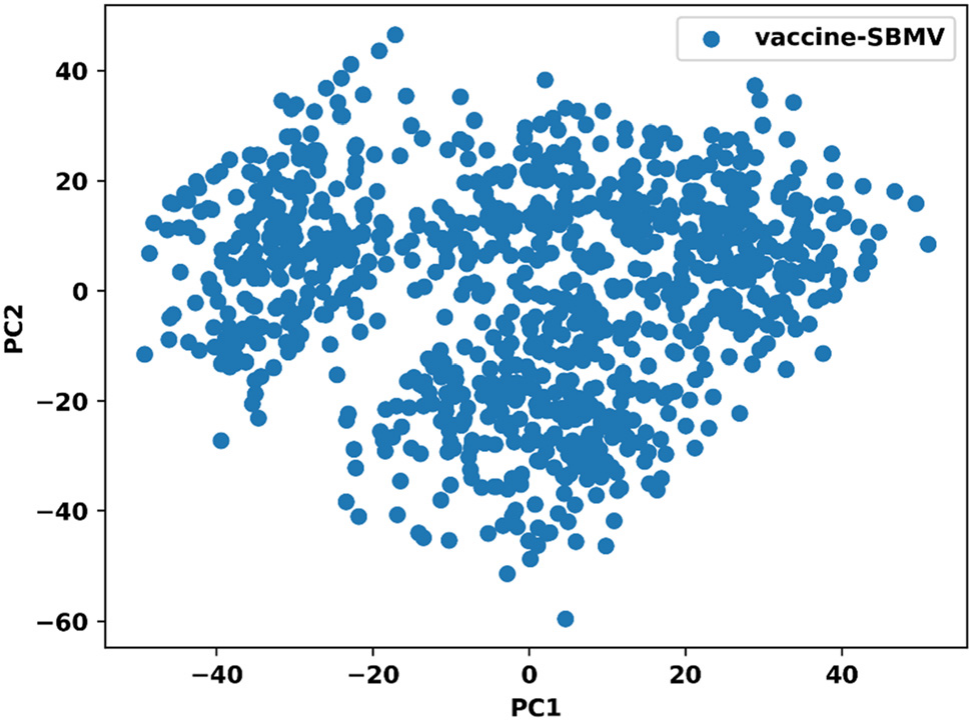
Principal component analysis plot of the vaccine–TLR4 complex showing the distribution of PC1 vs PC2 during the simulation.

The structural dynamics of the vaccine–TLR4 complex during the molecular dynamics simulation were further examined using principal component analysis (PCA). The projection of the first principal component (PC1) against the second principal component (PC2) showed a broad distribution along both axes, with PC1 ranging approximately from −50 to +50 and PC2 from −60 to +50, as presented in Figure 12. This distribution reflects conformational variation sampled during the simulation. Several dense clusters were observed within the PCA plot, corresponding to frequently sampled conformational states of the vaccine–TLR4 complex. The clustering pattern indicates transitions between different structural conformations over the simulation period. These results describe the conformational space explored by the complex during the 100 ns trajectory.

### Dynamic cross-correlation matrix

**Figure 13:**
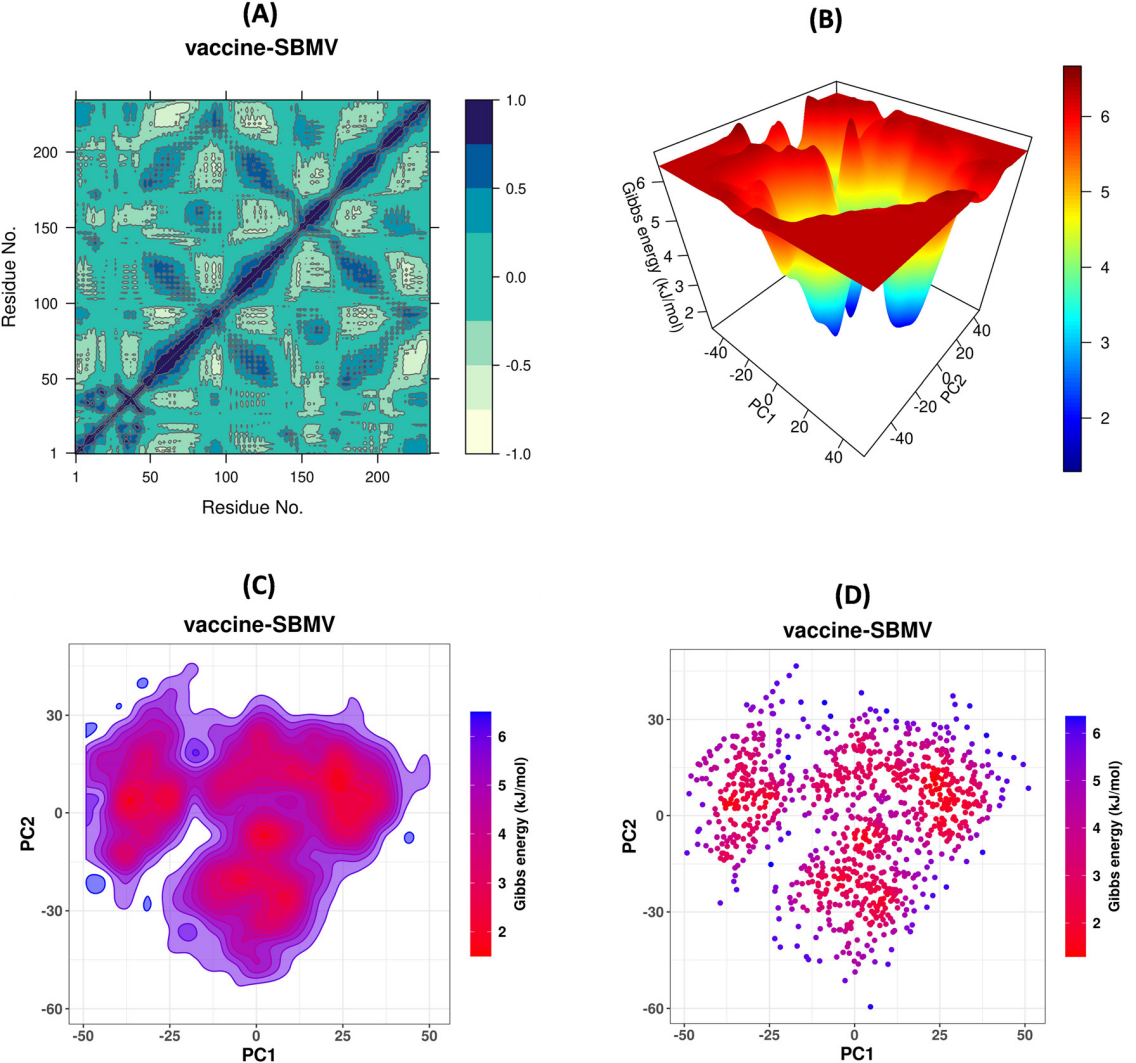
Conformational and energetic analyses of the vaccine–TLR4 complex. **(A)** DCCM plot showing correlated and anti-correlated residue motions. **(B)** Three-dimensional free energy landscape illustrating global and local energy minima. **(C)** Two-dimensional free energy contour map in PC1–PC2 space. **(D)** Gibbs free energy scatter plot showing the distribution of conformations based on PC1 and PC2 coordinates.

The dynamic cross-correlation matrix analysis was performed to evaluate correlated and anti-correlated motions between residue pairs during the simulation. The correlation coefficients ranged from −1.0 to +1.0. In the vaccine–TLR4 complex, strong positive correlations (approaching +1.0) were observed along the diagonal, indicating coordinated intra-domain movements. Moderate anti-correlated motions (ranging from −0.4 to −0.7) were detected in off-diagonal regions, especially between residues 50–150 and 180–230, as shown in Figure 13A. These correlation patterns reflect the dynamic relationships between different regions of the complex during the simulation.

### Free energy landscape

The global conformational energy profile of the complex was analyzed using principal components PC1 and PC2 to construct the three-dimensional free energy landscape (3D-FEL). The plot showed a deep global minimum at approximately PC1≈−5 and PC2≈−10, corresponding to a Gibbs free energy of approximately 2.0 kJ/mol. Additional local minima were observed across the landscape, with energy values approaching 6.5 kJ/mol (Figure 13B). The two-dimensional free energy landscape (2D-FEL) provided a projection of the conformational energy distribution in PC1–PC2 space. The lowest energy regions were primarily located within PC1 values of approximately −15 to +10 and PC2 values of approximately −10 to +5, with free energy minima near 2.0 kJ/mol (Figure 13C). Higher energy regions extended toward approximately 6.5 kJ/mol. The scatter plot of individual conformations, color-coded by Gibbs free energy, further illustrated the distribution of conformers across PC1 and PC2 coordinates. Dense clusters of low-energy conformations (2.0–3.0 kJ/mol) were concentrated around PC1≈−10 and PC2≈−10, whereas higher-energy conformations were more sparsely distributed (Figure 13D). These results describe the conformational states sampled by the complex during the simulation.

### Codon optimization and *In silico* cloning

**Figure 14:**
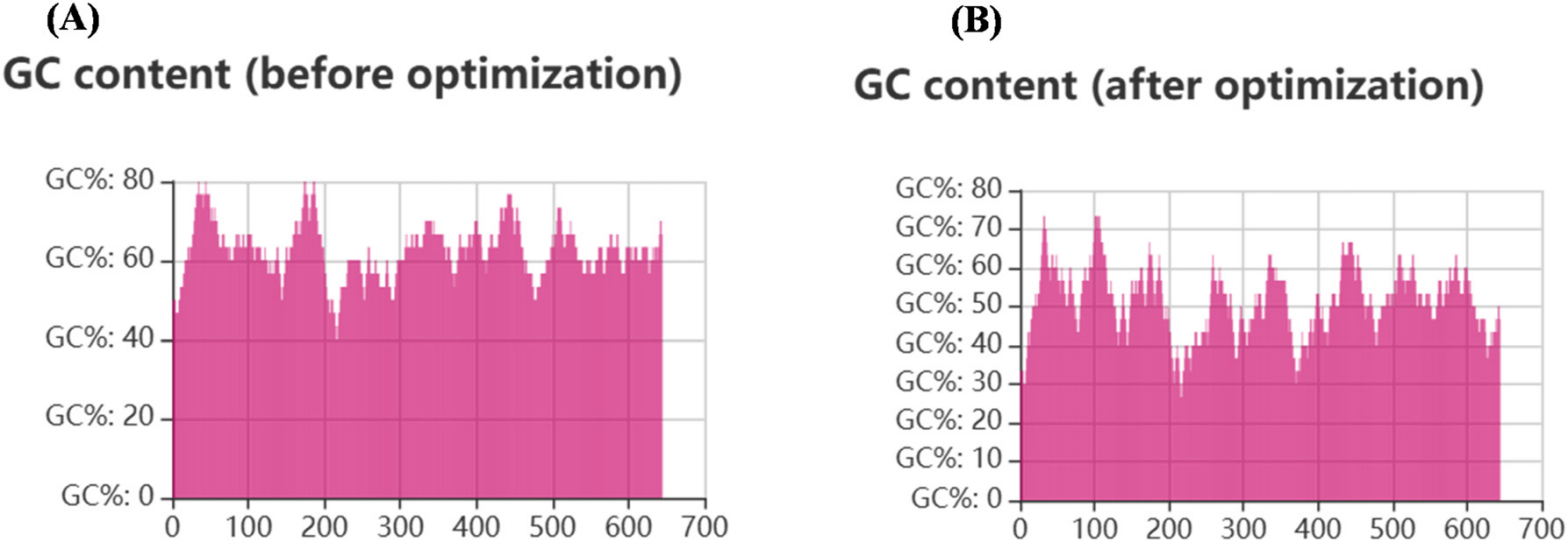
GC content distribution before and after codon optimization (A) GC content before codon optimization. (B) GC content after codon optimization.

**Figure 15:**
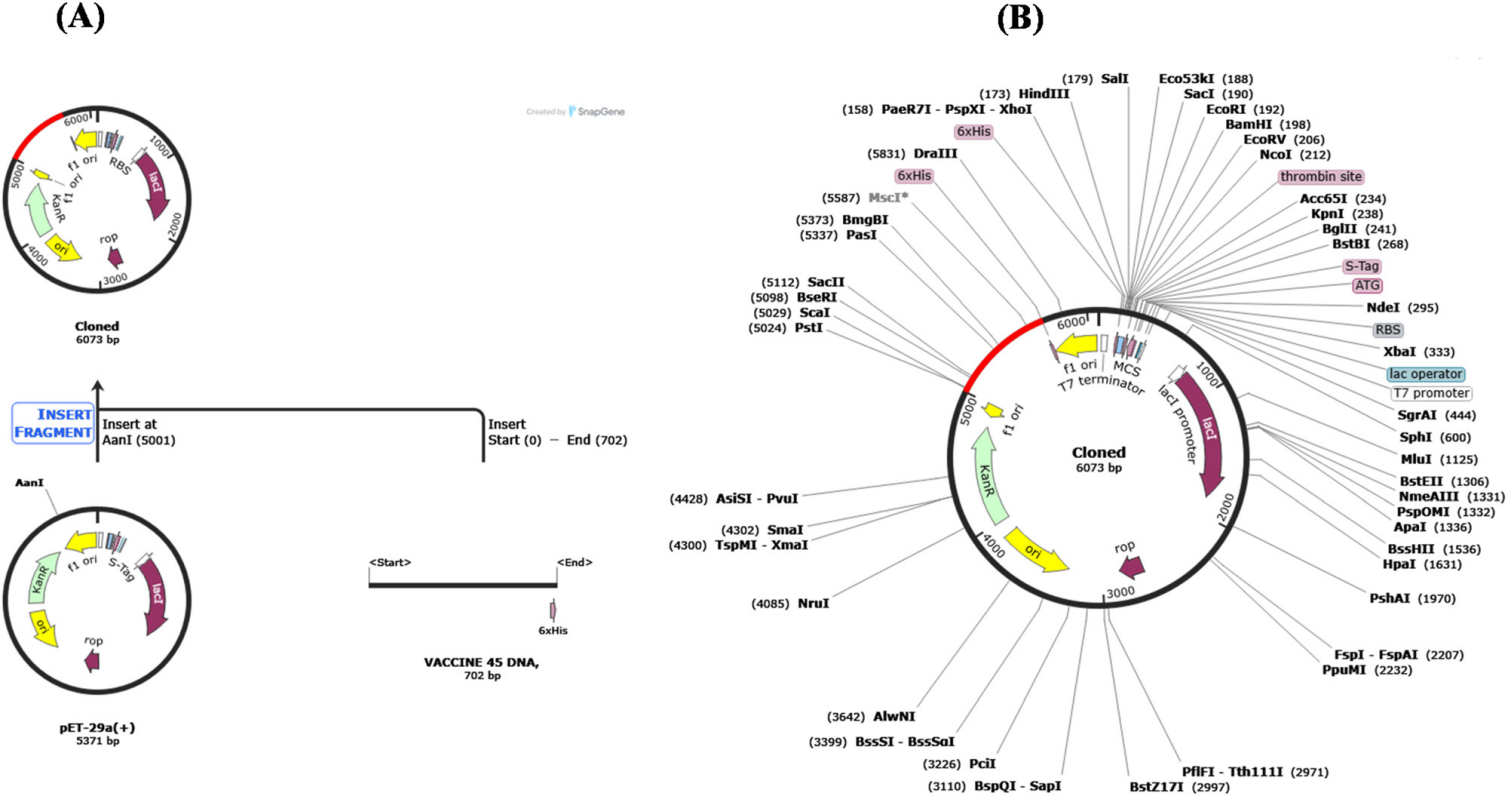
*In silico* cloning of the vaccine construct. **(A)** Cloning workflow. **(B)** Recombinant plasmid map showing restriction sites and inserted vaccine sequence.

Codon optimization was performed to enhance the expression of the construct in the selected host system. After optimization, the codon adaptation index increased slightly from 0.77 to 0.79, indicating improved compatibility with the host codon usage pattern. In addition, the GC content decreased from 62.54 % (Figure 14A) to 50.43 % (Figure 14A), resulting in a balanced GC composition that is suitable for efficient transcription and translation. For *in silico* cloning, the optimized DNA sequence of the vaccine construct (702 base pairs) was inserted into the pET-29a(+) expression vector (5,371 bp) using SnapGene software (Figure 15A). The vector was linearized using the restriction enzyme AanI at position 5001, and the vaccine insert was ligated into the vector. The final recombinant plasmid construct had an approximate size of 6,073 bp, reflecting the successful insertion of the 702 bp vaccine sequence, as illustrated in Figure 15B.

### Immune simulations

**Figure 16:**
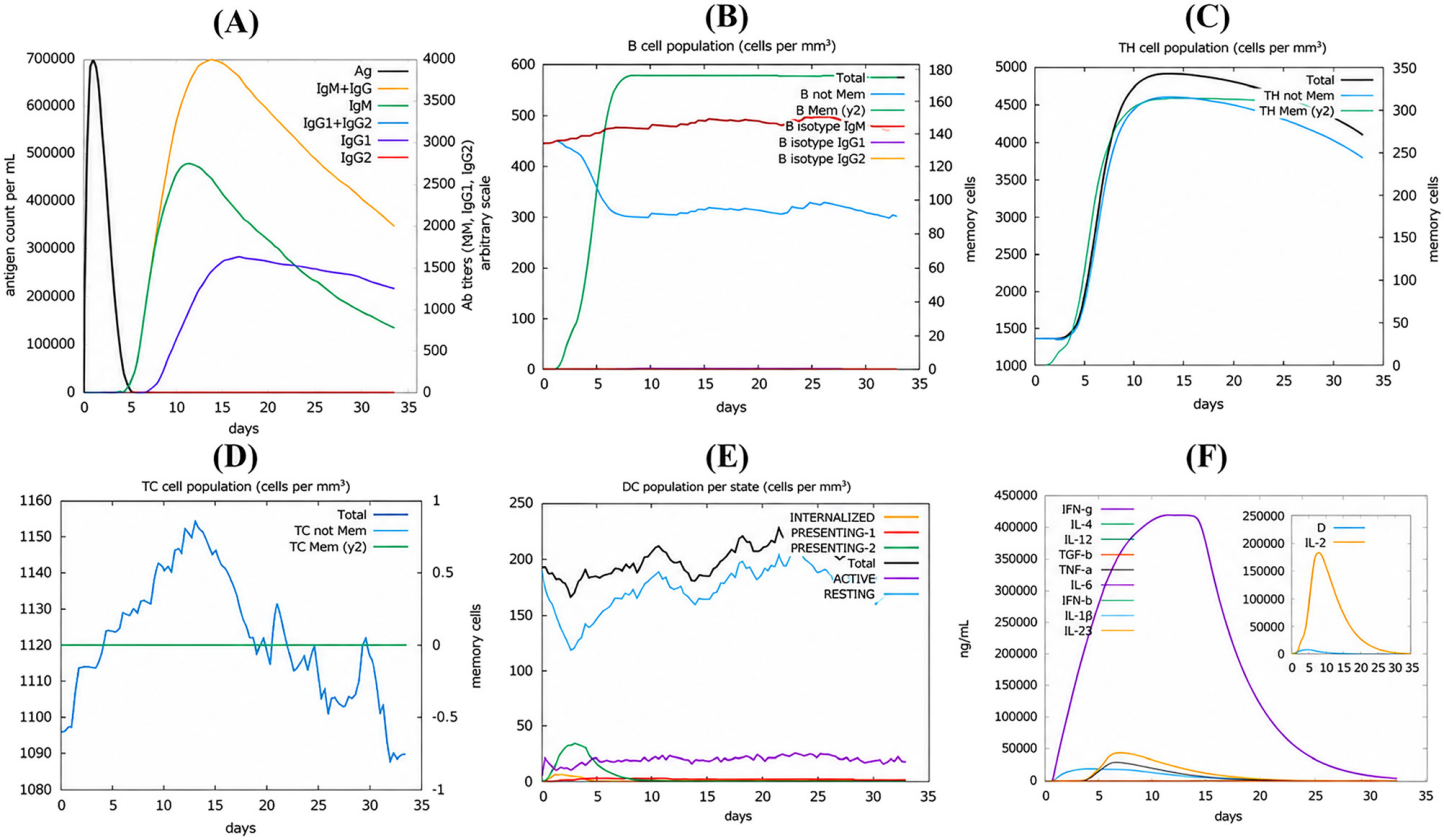
Immune simulation results of the designed multiepitope vaccine against *Streptobacillus moniliformis* **(A)** antigen and immunoglobulin levels. **(B)** B-cell population. **(C)** CD4^+^ T-helper lymphocyte count. **(D)** CD8^+^ cytotoxic T-lymphocyte count. **(E)** Dendritic cell population. **(F)** Cytokine and interleukin concentrations.

The immune response simulation of the designed multiepitope vaccine against *S. moniliformis* demonstrated dynamic immune activation patterns. The antigen concentration reached approximately 700,000 counts/mL on day 1 and declined to baseline levels by day 6, indicating antigen clearance during the simulated period (Figure 16A). An initial increase in IgM levels was observed, followed by elevated IgG responses, particularly IgG1 and IgG2 around day 13, reflecting immunoglobulin class switching during the simulation (Figure 16A). The B-cell population increased during the simulation period, while memory B-cell counts also increased, indicating the generation of immunological memory (Figure 16B). T helper (TH) cells showed a rapid increase during the simulation period, with memory TH-cell populations remaining stable during the later stages of the simulation (Figure 16C). Cytotoxic T-cell (TC) populations exhibited moderate fluctuations during the simulation period, maintaining a relatively stable overall trend with the presence of memory TC cells (Figure 16D). Dendritic cells demonstrated increased activity between days 3 and 6, corresponding to antigen presentation phases, with cell counts ranging from 150 to 200 cells/mm^2^ during the stimulation period (Figure 16E). Cytokine analysis revealed elevated IFN-γ and IL-2 responses during peak immune activation phases, whereas IL-10 and IL-6 were detected at comparatively lower levels (Figure 16F). Overall, the simulation results describe coordinated humoral and cellular immune response patterns following vaccine administration, as reflected by antigen clearance, antibody production, immune cell expansion, and cytokine expression trends.

## Discussion

In the present study, we designed and computationally evaluated a multiepitope-based vaccine candidate against *S. moniliformis* and found that the construct demonstrated favorable antigenicity, structural stability, broad population coverage, and predicted immune receptor interactions. Immune simulation further indicated coordinated humoral and cellular immune responses, supporting the potential immunogenicity of the designed vaccine. These findings address the research question of whether an immunoinformatics-guided multiepitope approach can generate a stable and immunogenic vaccine candidate against rat-bite fever.

*S. moniliformis* is a rod-shaped, Gram-negative bacterium and a recognized causative agent of rat-bite fever (RBF), a zoonotic infection commonly associated with exposure to infected rodents [47]. RBF remains a public health concern, particularly in regions with close human–rodent interactions, such as North America and parts of Asia [13]. The disease presents with systemic manifestations including fever, rash, and migratory polyarthralgia, and severe untreated infections may lead to invasive systemic complications [48]. Despite its clinical relevance, no licensed vaccine is currently available, and treatment depends largely on antibiotics, highlighting the need for alternative preventive strategies. Multiepitope vaccines have been widely explored for inducing targeted immune responses and improving antigen-specific immunogenicity [49]. Similar approaches have been explored against pathogens such as *Helicobacter pylori* [50], *Vibrio cholerae* [51], *Pseudomonas aeruginosa* [52], breast cancer [53], and *Aeromonas hydrophilla* [54]. In the present study, antigenic epitopes were selected from key proteins and screened for non-allergenicity and non-toxicity prior to vaccine construction. Epitope identification is considered a critical step in rational vaccine design [55]. Molecular docking and molecular dynamics simulations were performed to evaluate structural stability and receptor interaction profiles, providing insight into the predicted behavior of the vaccine construct within the immune environment [56], 57].

Importantly, molecular docking analysis was performed with Toll-like receptor 4 (TLR4) because it plays a central role in recognizing lipopolysaccharides from Gram-negative bacteria [58]. Activation of TLR4 triggers MyD88-and TRIF-dependent signaling pathways, leading to NF-κB activation and the production of pro-inflammatory cytokines that connect innate and adaptive immune responses [59]. The inclusion of beta-defensin-3 as an adjuvant further supports this approach, as beta-defensin-3 has been associated with modulation of TLR4-related immune signaling pathways [60]. Although other Toll-like receptors such as TLR2 and TLR5 contribute to bacterial recognition, TLR4 remains a major receptor involved in Gram-negative bacterial immune signaling [61].

Advances in immunoinformatics enabled the identification and prioritization of MHC class II epitopes, which play a central role in activating CD4+ T helper cells and coordinating adaptive immune responses. Two epitopes were selected from each protein, and associated HLA alleles were used for population coverage analysis. Selected epitopes met antigenicity criteria (>0.4) and were predicted to be non-allergenic and non-toxic. Human leukocyte antigen (HLA) class II molecules play an essential role in antigen presentation and adaptive immune regulation [62].

Physicochemical evaluation indicated that the vaccine construct possesses characteristics compatible with stable expression and structural integrity. The instability index was calculated as 32.50, suggesting favorable protein stability. Secondary structure prediction revealed 48.29 % alpha-helix, 14.53 % beta-strand, and 37.18 % coil regions. Ramachandran plot analysis showed that 97.6 % of residues were located in favored regions, and the ERRAT quality score of 96.789 supported acceptable structural geometry. Molecular docking demonstrated favorable interaction patterns with TLR4, including hydrogen bonds and interfacial contacts. Codon optimization improved the Codon Adaptation Index from 0.77 to 0.79, indicating enhanced compatibility for expression in *E. coli*. Although the computational findings are encouraging, *in silico* predictions require experimental validation. Further *in vitro* and *in vivo* studies are necessary to confirm immunogenicity, safety, and protective efficacy before clinical application.

### Limitations

Despite the promising findings, several limitations should be acknowledged. The vaccine design and evaluation were conducted entirely using computational and immunoinformatics approaches. Although these tools provide valuable predictive insights, they may not fully reflect the complexity of biological immune responses *in vivo*. Therefore, experimental validation through *in vitro* and animal model studies is necessary to confirm immunogenicity, safety, and protective efficacy. Molecular docking and molecular dynamics simulations were performed primarily using TLR4 as the target immune receptor. While TLR4 is highly relevant for Gram-negative bacterial recognition, evaluation with additional immune receptors could provide further insight into host immune recognition mechanisms. Furthermore, codon optimization and *in silico* cloning predict efficient expression in *E. coli*; however, actual protein expression, folding behavior, and biochemical stability require laboratory confirmation. Overall, additional experimental investigations are necessary before translating this computational vaccine candidate into a clinically applicable preventive strategy.

## Conclusions

This study highlights the potential of immunoinformatics-driven strategies for accelerating vaccine development against neglected zoonotic pathogens such as *S. moniliformis*. In the absence of an approved vaccine for rat-bite fever and amid increasing concerns regarding antimicrobial resistance, computational vaccine design offers a rational and efficient framework for identifying promising vaccine candidates. The proposed multiepitope construct represents a foundation for further experimental investigation. Future *in vitro* and *in vivo* studies will be essential to validate its immunogenicity, safety, and protective efficacy. Overall, this work contributes to the growing application of computational vaccinology as a supportive platform for the development of next-generation preventive strategies against emerging and underexplored bacterial infections.
